# Supplementary material for: Aeromonas trota Is Highly Refractory to Acquire Exogenous Genetic Material
Source: Microorganisms. 2024 May 28;12(6):1091. doi: 10.3390/microorganisms12061091 (PMC11206119; doi:10.3390/microorganisms12061091)
Supplement: Supplementary file 1 [file microorganisms-12-01091-s001.zip › microorganisms-3012996-supplementary.pdf]

Aeromonas trota is highly refractory to acquire exogenous genetic material.

Supplementary material

Table S1. Primers used in this study.

Primer	Sequence 5′-3′	Size (nt)	Tm (°C)	Reference
**Kan F2**	CCATATTCAGCGTGAAACGA	20	52.9	This study
**Kan R2**	GCCCAGGCAGTTCCACAGAA	20	60.3	
**pRANGER_F1**	AGAGGTTCCAACTTTCACCAT	20	54.1	
**CmR_R2**	TCATTAAGCATTCTGCCGACA	21	54.6	
**ERIC-1**	ATGTAAGCTCCTGGGGATTCAC	22	56.7	[37]
**ERIC-2**	AAGTAAGTGACTGGGGTGAGC	21	56.7	
**gyrA2F**	ATGAGCGATCTGGCCAGAGA	20	58.2	[33]
**gyrA9R**	CGCGCCTTGTTCACCTGATA	20	57.7	
**gyrB3F**	TCCGGCGGTCTGCACGGCGT	20	69.4	
**gyrB14R**	TTGTCCGGGTTGTACTCGT	19	56.1	
**rpoD70Fs**	ACGACTGACCCGGTACGCATGTA	23	62	
**rpoD11R**	ATGCTCATGCGRCGGTTGAT	20	58.8	

Table S2. GenBank accession numbers of the reference strains sequences.

Strains	*gyr*A	*gyr*B	*rpo*D
A. allosaccharophila	HQ443156.1	AJ868385.1	AY169348.1
A. aquatica	HG970916.1	HG970927.1	HG970949.1
A. aquatilis	LT630730.1	LT630723.1	LT630716.1
A. australiensis	NZ_CDDH01000006.1	NZ_CDDH01000074.1	NZ_CDDH01000001.1
A. bestiarum	NZ_JPWL01000008.1	JPWL01000007.1	NZ_JPWL01000072.1
A. bivalvium	NZ_CDBT01000004.1	NZ_CDBT01000055.1	NZ_CDBT01000016.1
A. cavernicola	HQ443164.1	HQ442702.1	HQ442864.1
A. caviae	CDBK01000094.1	NZ_CDBK01000069.1	NZ_CDBK01000071.1
A. crassostreae	LT630726.1	LT630719.1	LT630712.1
A. dhakensis	NZ_CDBP01000029.1	NZ_CDBP01000023.1	NZ_CDBP01000072.1
A. diversa	NZ_CDCE01000034.1	NZ_CDCE01000027.1	NZ_CDCE01000007.1
A. encheleia	NZ_CDDI01000011.1	NZ_CDDI01000004.1	NZ_CDDI01000017.1
A. enterica	LT630725.1	LT630718.1	LT630711.1
A. eucrenophila	NZ_CDDF01000001.1	NZ_CDDF01000006.1	NZ_CDDF01000016.1
A. finlandiensis	HG970912.1	HG970923.1	HG970945.1
A. fluvialis	NZ_CDBO01000034.1	NZ_CDBO01000069.1	NZ_CDBO01000043.1
A. hydrophila	HQ443174.1	JN711776.1	JN215537.1
A. intestinalis	LT630724.1	LT630717.1	LT630710.1
A. jandaei	NZ_CDBV01000025.1	NZ_CDBV01000026.1	AY169341.1
A. lacus	HG970914.1	HG970925.1	HG970947.1
A. lusitana	NZ_PGCP01000021.1	NZ_PGCP01000011.1	NZ_PGCP01000041.1
A. media	NZ_CDBZ01000214.1	NZ_CDBZ01000214.1	NZ_CDBZ01000214.1
A. molluscorum	HQ443110.1	EF465521.1	EF465515.1
A. paramedia	NZ_JAAROG010000011.1	NZ_JAAROG010000027.1	NZ_JAAROG010000001.1
A. piscicola	NZ_CDBL01000018.1	NZ_CDBL01000090.1	NZ_CDBL01000080.1
A. popoffii	NZ_CDBI01000024.1	NZ_CDBI01000062.1	NZ_CDBI01000001.1
A. rivipollensis	NZ_CP027856.1	NZ_CP027856.1	NZ_CP027856.1
A. rivuli	NZ_CDBJ01000054.1	NZ_CDBJ01000087.1	NZ_CDBJ01000065.1
A. salmonicida	NZ_CDDW01000106.1	NZ_CDDW01000008.1	NZ_CDDW01000026.1
A. sanarellii	NZ_CDBN01000004.1	NZ_CDBN01000092.1	NZ_CDBN01000049.1
A. schubertii	NZ_CDDB01000010.1	NZ_CDDB01000021.1	NZ_CDDB01000081.1
A. simiae	NZ_CDBY01000023.1	NZ_CDBY01000061.1	NZ_CDBY01000062.1
A. sobria	NZ_CDBY01000023.1	NZ_CDBY01000061.1	NZ_CDBY01000062.1
A. taiwanensis	NZ_BAWK01000039.1	NZ_BAWK01000086.1	NZ_BAWK01000094.1
A. tecta	NZ_CDCA01000010.1	NZ_CDCA01000023.1	NZ_CDCA01000028.1
A. trota	NZ_CDCG01000018.1	NZ_CDCG01000031.1	NZ_CDCG01000026.1
A. veronii	HQ443160.1	AY101795.1	HQ442833.1
Oceanimonas sp.	CP003171.1	CP003171.1	CP003171.1

Table S3. GenBank accession number of the reference genomes used in phylogenomic, *is*DDH and ANI analysis.

Strain	Accession number
A. allosaccharophila FDAARGOS 933	NZ_CP065745.1
A. aquatica AE235	NZ_JRGL01000001.1
A. australiensis CECT 8023	NZ_CDDH01000001.1
A. bestiarum GA97-22	NZ_PPUX01000001.1
A. bivalvium ZJ19-2	NZ_NXBQ01000010.1
A. cavernícola DSM 24474	NZ_PGGC01000001.1
A. caviae NCTC12244	NZ_LS483441.1
A. caviae WP8-S18-ESBL-04	NZ_AP022254.1
A. caviae FIONA 16800	NZ_CP121794.1
A. caviae KAM643	NZ_AP026403.1
A. caviae BC11	NZ_CP102334.1
A. dhakensis TN14	NZ_LR963104.1
A. diversa CECT 4254	NZ_CDCE01000001.1
A. encheleia NCTC12917	NZ_LR134376.1
A. eucrenophila CECT 4224	NZ_CDDF01000001.1
A. finlandiensis 4287D	NZ_JRGK01000001.1
A. fluvialis LMG 24681	NZ_CDBO01000001.1
A. hydrophila FDAARGOS 916	NZ_CP065651.1
A. jandaei FDAARGOS 986	NZ_CP066092.1
A. lacus AE122	NZ_JRGM01000001.1
A. lusitana MDC 2473	NZ_PGCP01000001.1
A. media TR3 1	NZ_CP075564.1
A. molluscorum 848	NZ_AQGQ01000001.1
A. paramedia 3925	NZ_JAAROG010000001.1
A. piscicola LMG 24783	NZ_CDBL01000001.1
A. popoffii CIP 105493	NZ_CDBI01000001.1
A. rivipollensis KN-Mc-11N1	NZ_CP027856.1
A. rivuli 20-VB00005	NZ_CP079742.1
A. salmonicida AS3	NZ_CP110645.1
A. sanarellii LMG 24682	NZ_CDBN01000001.1
A. schubertii ATCC 43700	NZ_LPUO01000001.1
A. simiae A6	NZ_CP040449.1
A. sobria CECT 4245	NZ_CDBW01000001.1
A. taiwanensis LMG 24683	NZ_CDDD01000001.1
A. tecta CECT 7082	NZ_CDCA01000001.1
A. trota FDAARGOS 1510	NZ_CP083625.1
A. trota FDAARGOS 1459	NZ_CP082887.1
A. trota FDAARGOS 1537	NZ_CP084358.1
A. trota CECT 4487	NZ_CDCG01000001.1
A. trota FDAARGOS 1509	NZ_CP083626.1
A. trota FDAARGOS 1536	NZ_CP083612.1
A. veronii FDAARGOS 632	NZ_CP044060.1
Oceanimonas sp. GK1	CP003171.1

Table S4. Minimal inhibitory concentration of *A. trota*.

Strain	KAN	NAL	STR	CL	TET	DX	TMP
A. trota 3.23	<16	<16	<4	<1	<4	64	4
A. trota 3.25	<16	<16	<4	16	<4	64	4
A. trota 4.24	<16	<16	<4	<1	<4	64	2
A. trota 5.3	<16	<16	<4	<1	<4	64	4
A. trota 5.9	<16	<16	<4	<1	<4	64	1
A. trota 8.5	<16	<16	<4	<1	<4	<32	4
A. trota 9.1	<16	<16	<4	<1	<4	64	2
A. trota 9.2	<16	<16	<4	<1	<4	<32	2
A. trota 9.3	<16	<16	<4	<1	<4	64	1
A. trota 9.4	<16	<16	<4	<1	<4	128	2
A. trota 9.7	<16	<16	<4	<1	<4	256	2
A. trota 9.8	<16	<16	<4	<1	<4	64	2
A. trota 9.11	<16	<16	<4	<1	<4	64	1
A. trota 9.12	<16	<16	<4	<1	<4	64	2

Table S5. Features of assembled genomes.

Strain	GenBank accession No.	Raw sequences	Sequencing depth	Scaffolds No.	Genome size (Mb)	% G+C	CDS
A. caviae 6548	JAZDDN000000000	4,859,697	158.47	40	4.50	61.9	4081
A. trota 5.9	JAZDDQ000000000	4,563,869	152.13	20	4.38	59.9	4058
A. trota 9.1	JAZDDP000000000	4,662,125	155.40	26	4.41	59.8	4078
A. trota 9.3	JAVTII000000000	1,496,136	49.88	122	4.43	59.8	4115
A. trota 9.12	JAZDDO000000000	4,709,387	156.98	29	4.49	59.8	4217

Table S6. Features of prophages detected in assembled *Aeromonas* genomes.

Strain	Prophage	Length (Kb)	Prophage similarity (%)	% G+C
A. caviae 6548	1	39.3	PhiO18 (76.19)	59.57
	2	39.5	Lys12581Vzw (29.26)	58.28
	3	37.0	Fels-2 (16)	60.66
A. trota 9.1	1	38.8	Lys12581Vzw (32.35)	53.72
	1	37.1	PhiO18 (79.48)	56.63
A. trota 9.12	2	32.6	Lys12581Vzw (66)	53.57
	3	41.1	Mu (29.72)	57.16

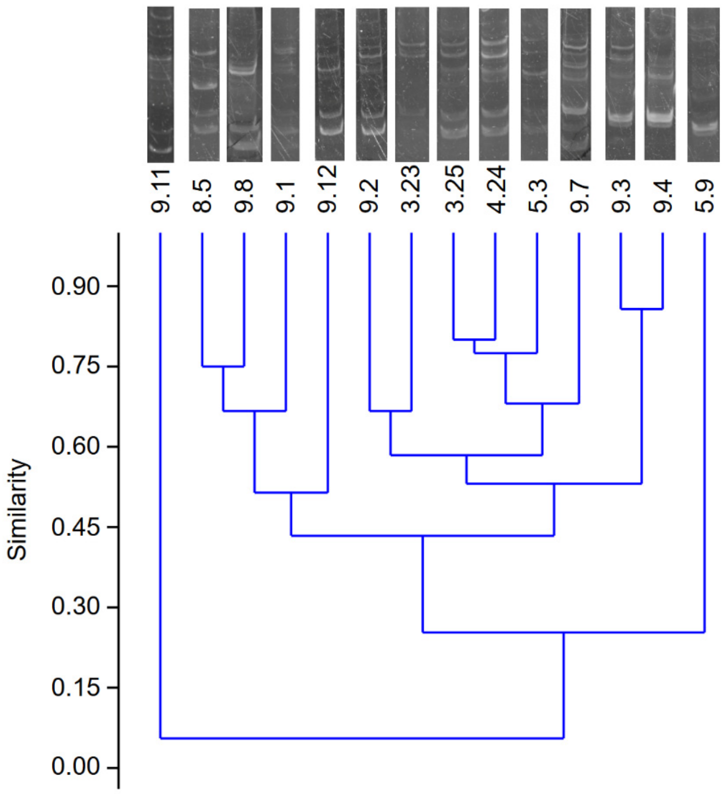

Figure S1. Dendrogram generated from enterobacterial repetitive intergenic consensus (ERIC-PCR) fingerprinting. Dendrogram constructed by UPGMA grouping method and Dice similarity coefficient using ERIC-PCR band patterns from 14 *A. trota* strains. Scale similarity values are from 0 to 1.0.

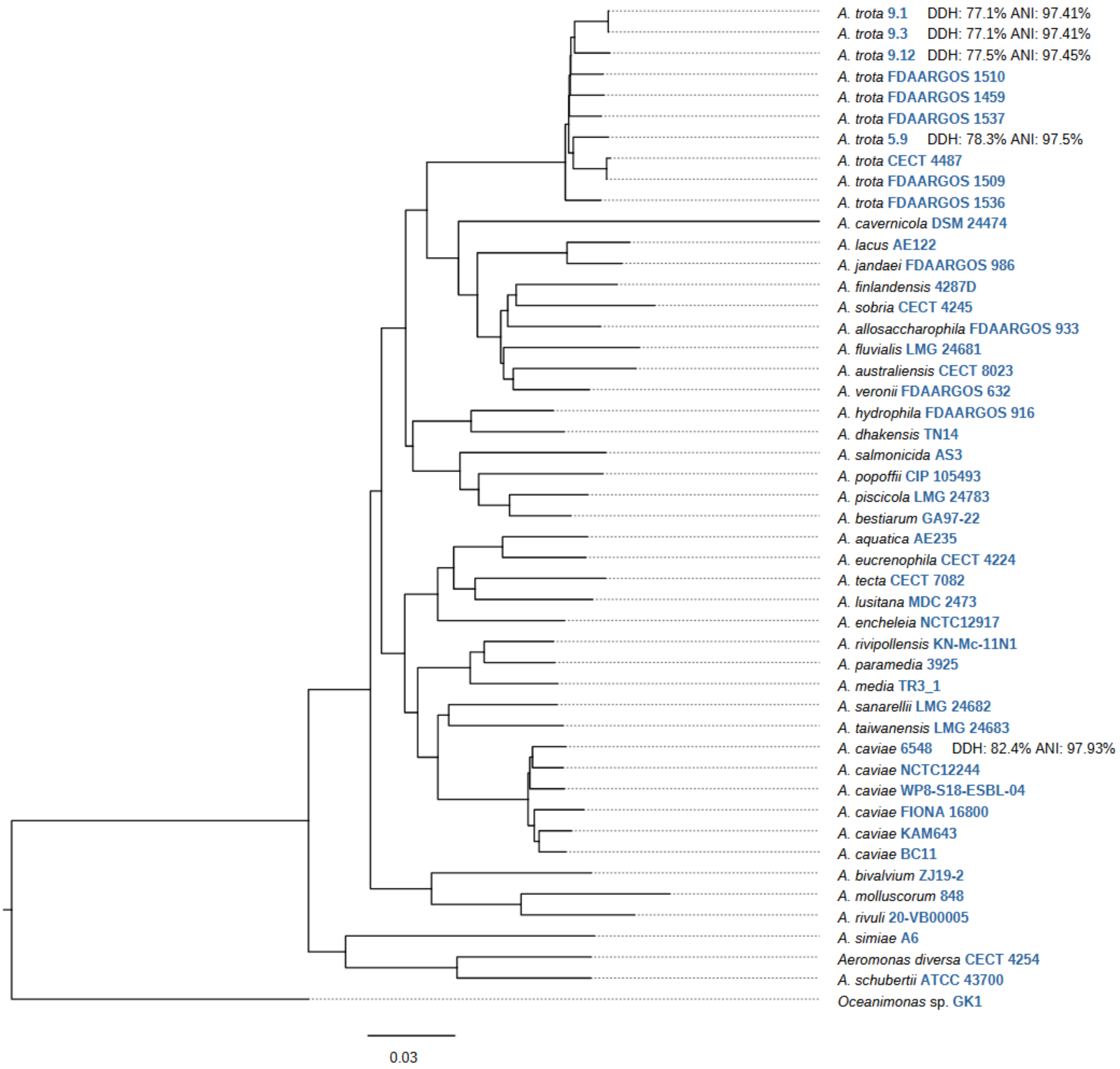

Figure S2. Phylogenomic analysis of *Aeromonas* spp. calculated by virtual genome fingerprints using VAMPhyRE software. Tree was constructed by Neighbor Joining method using available *Aeromonas* spp. genome sequences. DDH and ANI values of the sequenced *Aeromonas* genomes are indicated next to the strain name and indicate their respective relationship with *A. trota* or *A. caviae*.

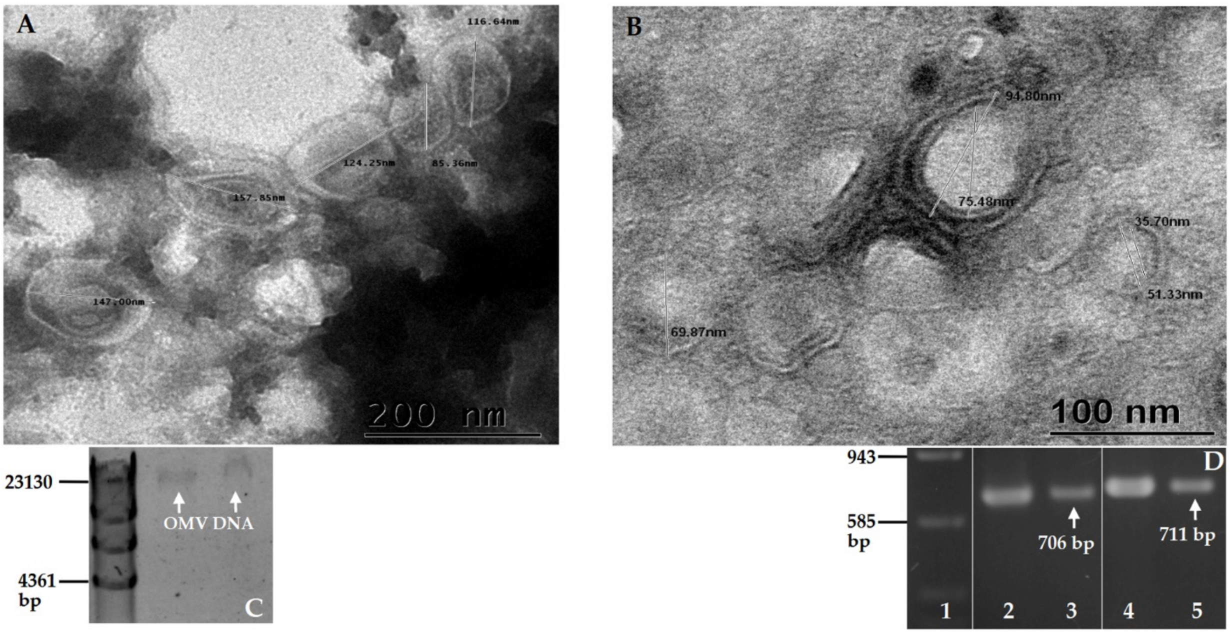

Figure S3. Outer membrane vesicles (OMV) characterization. A) OMV from donor *A. trota* 9.12 and B) *A. caviae* 6548. C) Whole DNA extracted from OMV. Lane 2: *A. trota* 9.12. Lane 3: *A. caviae* 6548. Lane 1: λ/HindIII. D) PCR amplification from OMV. Lane 2: *aph*A positive control. Lane 3: *aph*A from OMV. Lane 4: pRANGER BTB-3 positive control. Lane 5: pRANGER BTB-3 amplicon from OMV.
